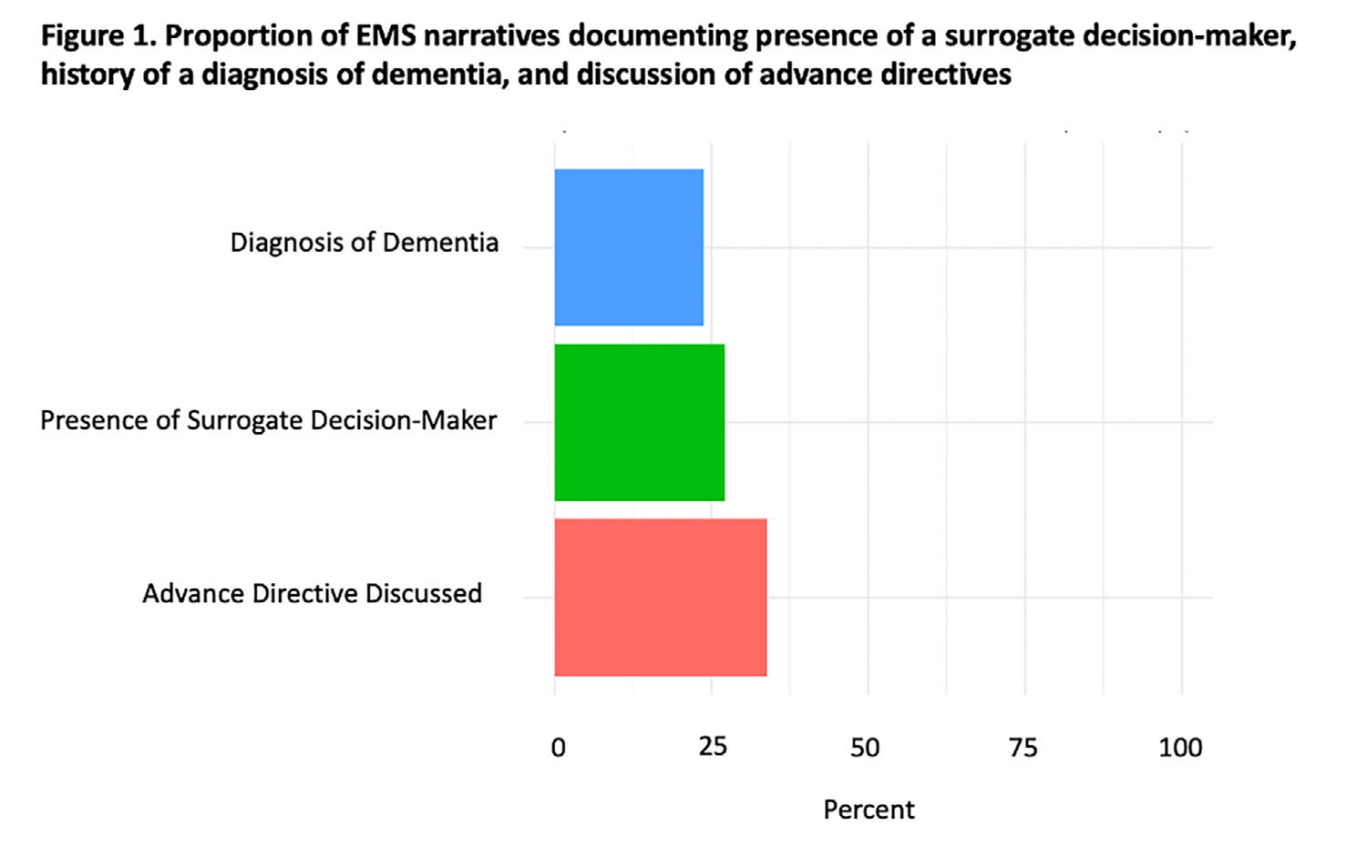# Prehospital treatment decisions for critically ill patients with dementia

**DOI:** 10.1002/alz.088613

**Published:** 2025-01-09

**Authors:** Lauren R Pollack, Jamie T Nomitch, Whitney Kiker, Ruth A Engelberg, Danae Dotolo, Lyndia Brumback, May Reed, Nicholas Johnson, Michael Sayre, Erin Kross

**Affiliations:** ^1^ University of Washington, Seattle, WA USA

## Abstract

**Rationale:**

Prior work has shown a preference among most people with dementia and their families for comfort‐focused care near the end‐of‐life. Nonetheless, intubation and mechanical ventilation are increasing over time without concurrent trends in improved survival, including among those with advanced dementia. A better understanding of prehospital decision‐making about intubation for people with dementia will guide efforts to increase goal‐concordant care at onset of critical illness.

**Methods:**

We identified eligible patients using the UW Medicine electronic health record (EHR) applying the following inclusion criteria: (1) age ≥ 55, (2) ICD‐code indicating dementia, (3) admission to a medicine service between 2011 and 2021, (4) treatment by paramedics, and (5) National Early Warning Score ≥7 indicating critical illness. We performed automated and manual abstraction of demographic and clinical variables from the EHR. We conducted a qualitative content analysis of emergency medical services (EMS) treatment narratives for patients with dementia, abstracting the number of cases in which there was documentation of: (1) the patient’s history of dementia, (2) presence of a surrogate decision‐maker, and (3) discussion of advance directives.

**Results:**

In a preliminary sample of 81 patients, the mean age was 80 years (SD 12) and 42% were female. About half (48%) were residing in a nursing home and 73% had clinical markers of advanced dementia. The mean Glasgow Coma Score at time of EMS assessment was 11 (SD 3.9). The most common documented reasons for EMS activation were respiratory complaints (30%), altered mentation (20%), and suspected sepsis (19%). Awareness of patients’ history of dementia was documented in about a third (31%) of cases, the presence of a surrogate decision‐maker in 21% of cases, and assessment of whether the patient had an advance directive in 22% of cases.

**Conclusion:**

Content analysis of EMS treatment narratives for critically ill patients with dementia suggests challenges faced by paramedics in eliciting patients’ treatment preferences, including having limited access to a surrogate decision‐maker with knowledge of the patient’s medical history, values and goals, and existing advance directives. Future work should elicit paramedic perspectives on challenges faced in providing goal‐concordant care for this patient population.